# Body weight variation predicts disease progression after invasive ventilation in amyotrophic lateral sclerosis

**DOI:** 10.1038/s41598-019-48831-9

**Published:** 2019-08-22

**Authors:** Yuki Nakayama, Toshio Shimizu, Chiharu Matsuda, Michiko Haraguchi, Kentaro Hayashi, Kota Bokuda, Masahiro Nagao, Akihiro Kawata, Kazuko Ishikawa-Takata, Eiji Isozaki

**Affiliations:** 1 ALS Nursing Care Project, Tokyo Metropolitan Institute of Medical Science, Tokyo, Japan; 2 Department of Neurology, Tokyo Metropolitan Neurological Hospital, Tokyo, Japan; 3 Department of Nutrition and Metabolism, National Institutes of Biomedical Innovation, Health and Nutrition, Tokyo, Japan

**Keywords:** Amyotrophic lateral sclerosis, Risk factors

## Abstract

Weight loss is an independent predictor of survival in the early stages of amyotrophic lateral sclerosis (ALS). However, the effects of weight variations on the functional prognosis after tracheostomy and invasive ventilation (TIV) in ALS remain unknown. This prospective cohort study aimed to investigate the relationship between weight loss before TIV and disease progression after TIV in ALS patients. Sixty ALS patients with TIV were enrolled and classified into subgroups based on the rate of decline in body mass index, from onset to TIV utilization (ΔBMI). During follow-up, we assessed the patients for presence of communication impairments, ophthalmoplegia, total quadriplegia, mouth opening disability, and dysuria. We analyzed the relationship between ΔBMI and the communication stage or motor disabilities. The log-rank test showed that patients with a ΔBMI ≥ 1.7 kg/m^2^/year showed a shorter period of preserved communication ability (*p* = 0.0001), shorter time to develop ophthalmoplegia (*p* = 0.0001), total quadriplegia (*p* < 0.0001), mouth opening disability (*p* < 0.0001), and dysuria (*p* = 0.0455). Cox multivariate analyses showed that a larger ΔBMI was an independent prognostic factor for the early development of ophthalmoplegia (*p* = 0.0400) and total quadriplegia (*p* = 0.0445). Weight loss in the early stages of ALS predicts disease progression in patients with advanced stages of ALS using TIV.

## Introduction

In amyotrophic lateral sclerosis (ALS), respiratory failure due to respiratory muscle paralysis or respiratory infection is a critical problem leading to death^1^. The median survival time of patients with ALS in Japan is 4 years from disease onset, for patients who do not utilize a ventilator^2^. Mechanical ventilation is effective in prolonging the survival of ALS patients beyond respiratory failure, while tracheostomy and invasive ventilation (TIV) may extend the survival time by several years or even more than a decade and maintain the patients’ quality of life^3–6^.

Several factors have been established as predictive factors for the prognosis of ALS, including age at onset, body region affected at onset, diagnostic delay from onset, progression rate of the revised ALS functional rating score (ALSFRS-R) from onset, and respiratory, bulbar, nutritional, and psychological factors. These factors were found to be related to survival^7–11^. Widespread emerging fasciculation potential, hyperexcitability of the peripheral motor axons, and hyperexcitability of the motor and sensory cortices are predictors of short-term survival in ALS^12–15^. With regard to the nutritional aspect, low body mass index (BMI) at diagnosis^16–18^, rapid body weight decline^19–21^, and hypermetabolism^22,23^ in the early stages typically indicate a short survival time or an earlier need for tracheostomy.

In contrast with the above early-stage prognostic factors, studies reporting the predictive factors for advanced stage ALS patients who have undergone TIV are limited^5,24^. Rapid progression from onset to TIV utilization predicts the development of severe communication impairments in advanced stages ALS patients using TIV. However, it remains unknown if weight loss prior to TIV use can be used as a long-term functional prognostic predictor. This study aimed to investigate the relationship between body weight variations in the early stages of ALS before TIV and disease progression in more advanced stages after TIV. This is the first study to demonstrate that a nutritional problem in ALS has a disease-specific role as a predictor not only for survival in non-TIV patients but also for disease progression in patients with long-term TIV.

## Methods

### Participants

**Figure 1:**
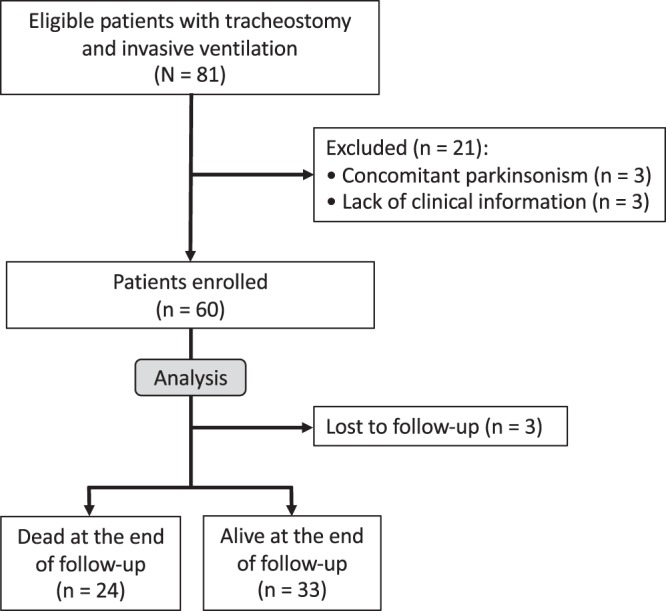
Flowchart of patient enrolment and selection of study sample.

Eighty-one patients with sporadic or familial ALS who utilized TIV were enrolled in this single-center, prospective open cohort study (Fig. 1). All patients were regularly followed up via home visits or regular admission to the Tokyo Metropolitan Neurological Hospital, Tokyo, Japan, between 2005 and 2017^5,24^. We included patients who had already been using TIV before 2005 and those who began to use TIV after 2005. All patients fulfilled the diagnostic criteria categorizing the disease as “clinically definite”, “clinically probable”, “clinically probable-laboratory supported”, or “clinically possible” ALS, according to the revised El Escorial criteria^25^. We also included patients with ALS of the progressive muscular atrophy type^26^. Of the 81 patients enrolled, three were excluded from the follow-up because of a concomitant Parkinsonism of unknown cause; another 18 were excluded because of incomplete assessment and lack of sufficient clinical information during follow-up. Sixty patients were finally enrolled in this study, three of whom had a family history of ALS, and two had superoxide dismutase 1 gene variants. ALS-related genes were evaluated only when the examination was possible owing to clinical implications and when allowed by the patients or their family. All patients showed a persistent and progressive disease course both before and after TIV use and showed severe generalized muscle atrophy along with tongue and respiratory muscle weakness. No other diseases affecting the nervous system were found during follow-up. Twenty-four patients died during the follow-up period, and three withdrew from the study over the 5-year period because they transferred to other hospitals. The study lasted until December 2017, and 33 patients survived at the end of the study (Fig. 1).

### Assessment

The neurological status, functional motor deficits, and ability to communicate (communication impairment stage) were evaluated in the patients during home visits by neurologists and nurses, and medical records were reviewed during regular admission to our hospital^5,24^. For patients who had already been using TIV before the study, clinical data before 2005 were retrospectively collected from the medical database. Of the 60 patients enrolled, none exhibited obvious dementia by routine neurological examinations throughout the follow-up period, although it was not possible to completely evaluate the cognitive and psychiatric functions of all the patients with advanced stages of ALS. All the patients showed normal orientation and reasonable conversation content.

To assess disease progression in ALS patients after TIV use, we assessed their communication ability according to our previously reported method^5,24,27^. The communication stage was defined using an augmentative and alternative communication device and classified into five stages as follows: stage I, communicated using sentences; stage II, one-word answers only; stage III, non-verbal yes/no responses; stage IV, occasional non-verbal yes/no response; and stage V, unable to communicate by any means. This staging method can be easily applied to TIV-ALS patients with severely deteriorating motor functions who cannot be fully assessed using conventional grading scales, such as ALSFRS-R or the Norris score^28,29^. Stage V corresponds to a totally locked-in state, in which all voluntary movements, including ocular movements, are lost^30,31^. The communication stage was prospectively and regularly assessed after initiation of TIV.

Furthermore, we evaluated the following four types of motor disabilities; obvious oculomotor limitations, total quadriplegia, mouth opening disability, and dysuria requiring urinary catheter insertion^5,24^. Obvious oculomotor limitations (ophthalmoplegia) were defined as persistently abnormal ocular movements during a bedside examination by at least two neurologists. The evaluation of ocular movements included examinations of slow pursuit movement and ocular saccade speed with a vertical and horizontal gaze. The most common abnormalities were vertical ophthalmoplegia and slow saccade (slow eye movement)^32–34^. Total quadriplegia indicated that even slightly visible muscle movements of the limbs were no longer possible. Inability to open mouth indicated that a patient was completely unable to move his/her jaw and open his/her mouth from a natural position. Dysuria indicated voiding difficulties that required placement of a permanent urinary catheter. The causes of dysuria (pelvic floor muscle weakness or neurogenic bladder) were not identified; however, all indications for urinary catheterization in individual patients were included for the analysis even though this procedure was only performed as a palliative care to reduce the patients’ and caregivers’ burden^35^. We defined the period of development of these manifestations during the follow-up period for each patient.

We also evaluated the following factors during the clinical assessment: sex, age at onset, body region affected at onset, height (m), disease duration from onset to initiation of TIV, and disease duration from TIV use to the final evaluation time. For assessment of the nutritional condition, we evaluated patients’ body weight (kg) at the time of diagnosis, at the time of TIV use, and at the end of follow-up. We calculated the BMI (kg/m^2^) at each point, and annual rate of BMI decline (ΔBMI) from the time of diagnosis to the time of TIV use, using the following formula: (BMI at the time of diagnosis–BMI at the time of TIV use)/time interval from diagnosis to TIV use (year)^20,21^. We classified the patients into two subgroups (those with ΔBMI < 1.7 kg/m^2^/year and those with ≥1.7 kg/m^2^/year) since the mean value of ΔBMI of the total patient cohort was 1.7 kg/m^2^/year. We also evaluated the energy intake (kcal/day) via enteral tube at the time of TIV use and at the end of follow-up. The energy intake of each patient was determined by clinicians according to patient’s nutritional condition including body weight and waist circumference.

### Statistical analysis

We used Welch’s t-test or chi-square test for data comparison between the two groups and Pearson’s correlation coefficient for correlation analysis. We compared the clinical data including the BMI values at each evaluation point, and the rate of occurrence of the four types of motor disabilities between the two subgroups. We also compared the time intervals from onset or TIV use to the time of progression from communication stage I to II, and the time intervals from onset to the time of appearance of the four motor disabilities between the subgroups. For time-to-event analyses from onset, the time required to progress from communication stage I to II and the time to the appearance of each of the motor disabilities were compared using the log-rank test, whereas Kaplan-Meier curves were used to estimate the absolute risk of events for each group. Finally, we performed a uni- and a multivariate analysis to determine the factors that were significantly associated with the length of time that the communication stage progression and four motor disabilities developed using the Cox proportional hazard model. In the multivariate analysis, we included the parameters that were significant in the univariate analysis. Statistical analyses were two sided, and *p* < 0.05 was considered significant. All analyses were performed using JMP for Macintosh version 13.0.0 (SAS Institute, Cary, NC, USA).

### Ethics statement

This study was approved by the ethics committees of the Tokyo Metropolitan Neurological Hospital (TS-H27-6, TS-H29-048, TS-H30-047) and the Tokyo Metropolitan Institute of Medical Science (no. 16–22). The study was performed in accordance with the ethical standards described in the latest revision of the Declaration of Helsinki, and in accordance with the Ethical Guidelines for Clinical Research of Tokyo Metropolitan Neurological Hospital and the Ethical Guidelines for Medical and Health Research Involving Human Subjects of Tokyo Metropolitan Institute of Medical Science. All patients or their legal guardians provided an informed consent before the study.

## Results

Table 1 outlines the clinical characteristics of the entire patient cohort and the patients in each subgroup. As previously mentioned, the mean value of the ΔBMI was 1.7 kg/m^2^/year. Comparing the two subgroups, patients with a ΔBMI ≥ 1.7 kg/m^2^/year showed a later onset, a higher frequency of bulbar onset, shorter duration from onset to TIV use, and larger BMI at the time of diagnosis. All patients received enteral nutrition via either nasogastric tube or gastrostomy tube. Enteral nutrition was initiated before TIV use in 36 patients, at the time of TIV use in 12 patients, and after TIV use in 12 patients. There was no difference in the timing of enteral nutrition between the two subgroups. The duration of enteral nutrition before TIV use was not different between the two subgroups (Table 1). Energy intake at the time of TIV was also not different. BMI at the end of follow-up was significantly larger than BMI at TIV use in a total cohort of patients and in both subgroups although the energy intake at the end of follow-up was decreased compared with that at TIV use for each cohort. There was no significant correlation between duration of enteral nutrition before TIV use and BMI at the time of TIV use (*p* = 0.6907). BMI increase after TIV until the end of follow-up was non-significantly larger in patients with a ∆BMI ≥ 1.7 kg/m^2^/year than in patients with a ∆BMI < 1.7 kg/m^2^/year (Table 1).

**Table 1:** Clinical characteristics of the patients.

	All	Subgroups classified by ΔBMI		*p* value
		ΔBMI < 1.7	ΔBMI ≥ 1.7	
Number of patients (n, %)	60	32 (53.3%)	28 (46.7%)	
Sex (male) (n, %)	43 (71.7%)	23 (71.9%)	20 (71.4%)	0.9695
Age at onset (year)	55.3 (11.9)	52.3 (12.5)	58.8 (10.4)	0.0341
Onset region (bulbar) (n, %)	14 (23.3%)	2 (6.3%)	12 (42.9%)	0.0005
Duration from onset to TIV use (year)	4.1 (3.4)	6.1 (3.7)	1.8 (0.8)	<0.0001
Duration of TIV use (m)	8.5 (6.0)	9.0 (6.5)	7.9 (5.3)	0.4543
BMI at diagnosis (kg/m^2^)	21.4 (2.7)	20.7 (2.9)	22.1 (2.3)	0.0473
BMI at TIV use (kg/m^2^)	17.3 (2.8)	17.7 (3.3)	16.9 (2.0)	0.2503
ΔBMI from onset to TIV use (kg/m^2^/year)	1.7 (1.7)	0.5 (0.6)	3.1 (1.5)	<0.0001
Duration of enteral nutrition before TIV use (year)	0.4 (1.2)	0.5 (0.2)	0.2 (0.5)	0.3113
Energy intake at TIV use (kcal)	1219 (267)	1221 (239)	1218 (298)	0.9648
Energy intake at the end of follow-up (kcal)	1049 (224)	1011 (212)	1091 (233)	0.1930
BMI at the end of follow-up (kg/m^2^)	19.2 (2.9)*	18.6 (2.9)^†^	19.8 (2.8)*	0.1309
BMI increase after TIV to the end of follow-up (kg/m^2^)	2.0 (3.0)	1.3 (3.0)	2.7 (2.8)	0.0690
Disabilities at the end of follow-up (n, %)				
Staying at communication stage I	25 (41.7%)	18 (56.3%)	7 (25.0%)	0.0131
Ophthalmoplegia	35 (58.3%)	15 (46.9%)	20 (71.4%)	0.0524
Total quadriplegia	32 (53,3%)	14 (43.7%)	18 (64.3%)	0.1101
Mouth opening disability	39 (65.0%)	18 (56.2%)	21 (75.0%)	0.1259
Dysuria with urinary catheter insertion	35 (58.3%)	19 (59.4%)	16 (57.1%)	0.8611

Values are expressed as number (%) or mean (SD). *p* values were obtained by comparing the values of the subgroups by using a chi-square test or Welch’s t-test. BMI at the end of follow-up was compared with BMI at TIV use using a paired t-test (*p < 0.0001 and ^†^*p* = 0.0233). TIV: tracheostomy and invasive ventilation, BMI: body mass index, ΔBMI: BMI decline rate.

At the end of the follow-up, patients with a ΔBMI ≥ 1.7 kg/m^2^/year showed a lower rate of staying at communication stage I than those with < 1.7 kg/m^2^/year (Table 1). The frequency of the development of ophthalmoplegia tended to be higher in patients with a ΔBMI ≥ 1.7 kg/m^2^/year.

Table 2 shows the duration from onset to the appearance of motor disabilities in the total patient cohort and the subgroups. Patients with a ΔBMI ≥ 1.7 kg/m^2^/year showed a shorter duration of staying at communication stage I from both onset and TIV use, and a shorter duration from onset to the appearance of ophthalmoplegia, total quadriplegia, mouth opening disability, and dysuria than patients with a ΔBMI < 1.7 kg/m^2^/year.

**Table 2:** Time from onset to development of motor disabilities.

	All	Subgroups classified by ΔBMI		*p* value
		ΔBMI < 1.7	ΔBMI ≥ 1.7	
Duration of stay at communication stage I from onset (year)	10.2 (7.5)	13.4 (8.5)	6.6 (4.0)	0.0002
Duration of stay at communication stage I from TIV use (year)	6.1 (5.2)	7.3 (6.0)	4.8 (3.7)	0.0494
Time from onset to ophthalmoplegia (year)	9.2 (7.3)	12.3 (8.5)	5.6 (2.8)	0.0002
Time from onset to total quadriplegia (year)	8.9 (6.4)	11.8 (7.1)	5.6 (3.0)	<0.0001
Time from onset to mouth opening disability (year)	9.7 (6.8)	12.5 (7.4)	6.6 (4.1)	0.0003
Time from onset to dysuria with urinary catheter insertion (year)	10.4 (7.4)	12.8 (8.4)	7.6 (4.8)	0.0039

Values are expressed as mean (SD). *p* values were obtained by comparing the values of the subgroups using Welch’s t-test. TIV: tracheostomy and invasive ventilation, ΔBMI: BMI decline rate.

**Figure 2:**
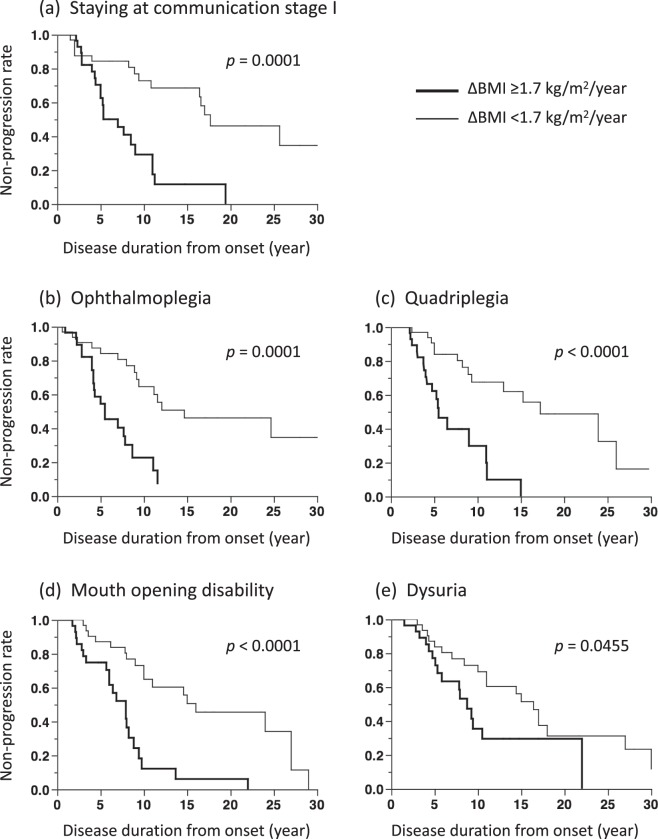
Comparison of the rate of staying at communication stage I from onset between patients with ΔBMI ≥ 1.7 kg/m^2^/year and < 1.7 kg/m^2^/year (**a**), and progression rate from onset to development of ophthalmoplegia (**b**), total quadriplegia (**c**), mouth opening disability (**d**) and dysuria with urinary catheter insertion (**e**). Kaplan-Meier analyses showed that patients with ΔBMI ≥ 1.7 kg/m^2^/year had a shorter period of staying at communication stage I (*p* < 0.0001), shorter periods to development of ophthalmoplegia (*p* < 0.0001), total quadriplegia (p < 0.0001), mouth opening disability (*p* < 0.0001) and dysuria (*p* = 0.0455) than patients with ΔBMI < 1.7 kg/m^2^/year.

Kaplan-Meier analyses showed that patients with a ΔBMI ≥ 1.7 kg/m^2^/year had shorter period of staying at communication stage I than those with a ΔBMI < 1.7 kg/m^2^/year (*p* = 0.0001, log-rank test; Fig. 2a). Kaplan-Meier analyses also showed that patients with a larger ΔBMI had shorter periods to development of ophthalmoplegia (*p* = 0.0001, log-rank test; Fig. 2b), total quadriplegia (*p* < 0.0001, log-rank test; Fig. 2c), mouth opening disability (*p* < 0.0001, log-rank test; Fig. 2d), and dysuria requiring urinary catheter insertion (*p* = 0.0455, log-rank test; Fig. 2e), than those with a lower ΔBMI.

Table 3 shows the results of uni- and multivariate analyses using the Cox proportional hazard model for period of staying at communication stage I from onset and periods from onset to development of each motor disability. For the univariate analyses, we examined the effect of sex, onset age, duration from onset to TIV use, duration of enteral nutrition before TIV use, duration of TIV use, and ΔBMI. All of these factors were analysed as dichotomized factors (Table 3). Univariate analyses showed that ΔBMI was significantly associated with the period of staying at communication stage I (*p* = 0.0002) and the periods to development of obvious ophthalmoplegia (*p* = 0.0002), total quadriplegia (*p* = 0.0001) and mouth opening disability (*p* < 0.0001). The duration of from onset to TIV use was also significantly associated with all the parameters for progression, while the duration of enteral nutrition before TIV had no effects on the progression. Multivariate analyses showed that the ΔBMI was significantly associated with the periods to development of ophthalmoplegia (*p* = 0.0400) and total quadriplegia (*p* = 0.0445).

**Table 3:** Uni- and multivariate Cox analyses of the prognostic factors for motor disabilities after TIV use.

		Univariate analysis		Multivariate analysis	
		HR (95% CI)	*p* Value	HR (95% CI)	*p* value
**Progression to communication stage II**					
Age at onset	≥65 vs. <65 years	6.73 (2.90–15.5)	<0.0001	6.80 (2.68–17.5)	<0.0001
Onset region	Bulbar vs. spinal	2.49 (1.10–5.26)	0.0292	0.45 (0.17–1.19)	0.1070
Duration from onset to TIV	<2 vs. ≥2 years	13.1 (5.21–36.7)	<0.0001	13.9 (3.91–57.6)	<0.0001
Duration of enteral nutrition before TIV use	<0.4 vs. ≥0.4 years	2.10 (0.97–5.23)	0.0621		
Duration of TIV use	≥5 vs. <5 years	0.64 (0.30–1.43)	0.2667		
ΔBMI	≥1.7 vs. <1.7 kg/m^2^/year	3.93 (1.90–8.42)	0.0002	1.72 (0.57–4.69)	0.3196
**Development of ophthalmoplegia**					
Age at onset	≥65 vs. <65 years	4.78 (2.12–10.4)	0.0003	4.71 (1.95–11.1)	0.0008
Onset region	Bulbar vs. spinal	1.29 (0.51–2.85)	0.5505		
Duration from onset to TIV use	<2 vs. ≥2 years	6.73 (2.82–16.9)	<0.0001	3.23 (1.18–9.83)	0.0224
Duration of enteral nutrition before TIV use	<0.4 vs. ≥0.4 years	1.63 (0.79–3.69)	0.1941		
Duration of TIV use	≥5 vs. <5 years	1.08 (0.51–2.50)	0.8448		
ΔBMI	≥1.7 vs. <1.7 kg/m^2^/year	3.96 (1.89–8.62)	0.0002	2.72 (1.01–6.96)	0.0400
**Development of total quadriplegia**					
Age at onset	≥65 vs. <65 years	2.31 (0.87–5.54)	0.0876		
Onset region	Bulbar vs. spinal	2.12 (0.80–5.08)	0.1222		
Duration from onset to TIV use	<2 vs. ≥2 years	8.11 (3.13–23.1)	<0.0001	4.44 (1.56–14.2)	0.0047
Duration of enteral nutrition before TIV use	<0.4 vs. ≥0.4 years	1.51 (0.72–3.48)	0.2837		
Duration of TIV use	≥5 vs. <5 years	2.13 (0.86–6.54)	0.1082		
ΔBMI	≥1.7 vs. <1.7 kg/m^2^/year	4.67 (2.11–10.9)	0.0001	2.75 (1.03–7.31)	0.0445
**Development of mouth opening disability**					
Age at onset	≥65 vs. <65 years	3.87 (1.75–8.16)	0.0013	3.46 (1.49–7.75)	0.0046
Onset region	Bulbar vs. spinal	3.14 (1.38–6.75)	0.0076	1.12 (0.44–2.81)	0.8045
Duration from onset to TIV use	<2 vs. ≥2 years	13.9 (5.45–38.9)	<0.0001	7.55 (2.40–26.1)	0.0008
Duration of enteral nutrition before TIV use	<0.4 vs. ≥0.4 years	1.24 (0.63–2.61)	0.5491		
Duration of TIV use	≥5 vs. <5 years	0.70 (0.33–1.60)	0.3866		
ΔBMI	≥1.7 vs. <1.7 kg/m^2^/year	4.34 (2.13–9.11)	<0.0001	2.27 (0.86–5.69)	0.0853
**Development of dysuria with urinary catheter insertion**					
Age at onset	≥65 vs. <65 years	2.32 (0.95–5.15)	0.0633		
Onset region	Bulbar vs. spinal	2.53 (1.09–5.41)	0.0319	1.19 (0.47–2.85)	0.6990
Duration from onset to TIV use	<2 vs. ≥2 years	6.81 (2.98–15.9)	<0.0001	6.38 (2.57–15.7)	<0.0001
Duration of enteral nutrition before TIV use	<0.4 vs. ≥0.4 years	1.44 (0.70–3.28)	0.3340		
Duration of TIV use	≥5 vs. <5 years	0.62 (0.28–1.49)	0.2729		
ΔBMI	≥1.7 vs. <1.7 kg/m^2^/year	2.02 (0.99–4.12)	0.0529		

## Discussion

Our study showed that the rate of BMI decline up to the time of TIV use was associated with communication impairments and progression of motor disabilities after TIV use in patients with ALS. Rapid BMI decline in the early stages may indicate severe functional deteriorations in the advanced stages of ALS. These results were in line with the findings of our previous study that showed that early rapid progression to the time of TIV use and the time of administering enteral nutrition were strongly associated with post-tracheostomy progression leading to failure to communicate^5^.

Studies on the functional rating of motor disabilities or disease staging after TIV use in ALS have been limited^5,24^. In such advanced stages, ALSFRS-R and Norris scores fall to almost zero and are not suitable for assessing motor disabilities. Oculomotor function and voiding ability, two abilities that are usually well preserved in the early stages of ALS, are often disturbed in the advanced stages after using TIV^5,24,31^. In particular, ALS patients who use TIV for more than 5 years frequently develop ophthalmoplegia, which may be followed by a totally locked-in state, dysuria requiring urinary catheter insertion, and various autonomic nervous manifestations such as hypertensive attacks and hypothermia^24,31,36^. Ophthalmoplegia is closely linked to communication impairments^5^. Based on this information, we adopted the communication staging and the four types of motor disabilities including ophthalmoplegia and dysuria in the present study. This staging system was recently developed to assess the communication ability of advanced ALS patients who utilized TIV^5,27^. Although it does not exactly reflect the grading or scoring of disease severity, this staging system has revealed the obvious heterogeneity of the clinical courses of ALS patients who use TIV long-term^5,24^.

The precise etiology of body weight loss in ALS still needs to be established^37^. Multiple factors may be involved in weight loss such as muscle wasting, dysphagia^19^, anorexia^38^, sympathetic hyperactivity^39^, respiratory burden, and hypermetabolism that may be specific to ALS^22,23,40,41^. Recent study reported that weight reduction rate accompanied by dysphagia was a strong predictor of short-term survival in ALS patients^19^, suggesting a pivotal role of both increased metabolic demand and decreased energy intake. Although the etiology of hypermetabolism also remains unknown, metabolic analyses using indirect calorimetry have revealed that about 40–50% of patients with ALS show hypermetabolism^22,23^. Recently, the involvement of the hypothalamus in the weight loss observed in ALS has been reported^42,43^. Inclusions of TDP-43 protein have been reported to accumulate in the hypothalamus in an ALS-model mouse^44^. A neuropathological study in patients also reported a TDP-43 pathology in the lateral hypothalamus that was significantly related to weight loss^45^. A morphometrical study using an MRI showed an atrophy of the hypothalamus in sporadic and familial ALS patients^46^. A clinical trial study in patients with ALS also showed the ineffectiveness of pioglitazone, which acts on the melanocortin pathway in the arcuate nucleus of the hypothalamus, suggesting a functional alteration of the hypothalamus in controlling appetite and body weight in ALS^47^.

The hypothalamic involvement in ALS indicates that ALS is a multisystem neurodegenerative disorder. The ophthalmoplegia and dysuria observed in our patients might be caused by involvement of the non-motor system including the frontal eye field and autonomic nervous centers in the brain^24,39^. Neuropathological evidences also support this idea^30,48,49^. Rapid body weight decline in the early stages of ALS might be the earliest non-motor manifestation due to multisystem neurodegeneration in ALS. Meanwhile, our clinical experience indicates that not all patients with ALS develop communication impairments or a “totally locked-in state” during TIV use^5,31^. Some patients with ALS remain in the communication stage I for more than 5 years after TIV use. The pathophysiological difference between patients who are able to communicate effectively and those who fail to do so is unknown, but it is possible that ALS consists of two disease categories exhibiting neurodegeneration limited to the motor neuron system or widespread multisystem neurodegeneration. This hypothesis is consistent with the neuropathological heterogeneity of distribution of TDP-43 immunoreactive inclusions^50^.

Although we could not control the patients’ energy intake before and after TIV use, there were no significant differences in the duration of enteral nutrition, energy intake, and BMI at the end of follow-up between the two subgroups (Table 1). As previously reported^21^, nutritional intervention in the early stage and resultant weight increase may improve survival before TIV use. However, if the hypermetabolism and weight loss at the early stage are associated with the hypothalamus lesion specific to ALS, one can easily imagine that early nutritional intervention will not easily modify the natural course of ALS with a long-term TIV use. Actually, the duration of enteral nutrition before TIV showed no effect on progression of motor disability after TIV use (Table 3). At the advanced stage with TIV, where many patients fall into marked muscle wasting with loss of respiratory burden and widespread brain atrophy including the hypothalamus and brainstem^51^, the metabolic demand would be markedly reduced even up to the level lower than the basal metabolic expenditure (e.g., up to 700–900 kcal/day)^52,53^, that is, “hypometabolism”. This idea is supported by the fact that our patients’ BMI was increased at the end of follow-up even if their energy intake was reduced (Table 1). Reactive energy consumption after enteral nutrition was reported to be completely lost in ALS patients in a totally locked-in state with TIV^53^, suggesting malfunction of central metabolic regulation by the hypothalamus^42^. We hypothesize that the hypothalamus lesion in ALS causes “hypermetabolism” with weight loss in the early stage and “hypometabolism” with weight gain in the advanced stage using TIV. The latter may lead to visceral fat accumulation^54^, macroglossia^55,56^, hyperosmolar hyperglycemic state^57^, hypertensive attack^36^, and hypothermia^24^.

This study has some limitations. First, the number of enrolled patients was small compared with those in previous clinical studies on ALS. However, as the study was only conducted in patients with long-term TIV use, the number of patients was not that small^4,54^. In Japan, 20–30% of patients with ALS use TIV^6,58^. The 81 patients in our study might correspond to 400 patients as a background population of ALS patients. Second, we could not define the premorbid baseline BMI in each patient. A rapid BMI decline from onset to the time of diagnosis is strongly associated with survival in ALS as well as a post-diagnostic BMI decline^19–21^. Third, we could not evaluate the effect of nutritional intervention in the early stages of ALS. Nutritional intervention has a potential effect on weight increase and may improve survival^21^. However, a nutritional therapy regimen has not yet been established, and a completely controlled prospective study for nutritional intervention would be difficult to perform. Similarly, it remains unknown whether nutritional intervention during TIV use is effective on the functional prognosis of ALS. Fourth, we did not evaluate the cognitive function of all patients. Although we excluded patients with obvious dementia, patients who lost ability to communicate might have unmeasurable cognitive decline. This issue should always be taken into consideration when conducting a clinical study on ALS patients with long-term TIV use.

Clinical research involving ALS patients with long-term TIV use is limited. Our findings may provide not only insight on the pathophysiology of ALS but also provide further information for patients and clinicians particularly at the time of decision-making for TIV use. Considering the quality of life of ALS patients who will survive for several years with TIV use, real evidence should be presented to patients before TIV use. Furthermore, patients should be informed of the possibility to fall into a totally locked-in state when TIV is used long-term. A considerable number of patients are afraid to lose their ability to communicate after TIV use^59^. Our findings may bring hope to patients who showed relatively slow progression and slow BMI decline before TIV use.

## Data Availability

Anonymized data not published within the article will be shared upon reasonable request from a qualified investigator.
